# Isomaltooligosaccharide-binding structure of *Paenibacillus* sp. 598K cycloisomaltooligosaccharide glucanotransferase

**DOI:** 10.1042/BSR20170253

**Published:** 2017-04-28

**Authors:** Zui Fujimoto, Naomi Kishine, Nobuhiro Suzuki, Ryuichiro Suzuki, Daiki Mizushima, Mitsuru Momma, Keitarou Kimura, Kazumi Funane

**Affiliations:** 1 Advanced Analysis Center, National Agriculture and Food Research Organization (NARO), 2-1-2 Kannondai, Tsukuba, Ibaraki 305-8602, Japan; 2 Biomolecular Research Unit, National Institute of Agrobiological Sciences, 2-1-2 Kannondai, Tsukuba 305-8602, Japan; 3 Department of Biological Production, Faculty of Bioresource Sciences, Akita Prefectural University, Shimoshinjyo-Nakano, Akita 010-0195, Japan; 4 Division of Food Biotechnology, Food Research Institute, National Agriculture and Food Research Organization, 2-1-12 Kannondai, Tsukuba 305-8642, Japan; 5 Department of Planning and General Administration, Institute of Crop Science, National Agriculture and Food Research Organization, 2-1-2 Kannondai, Tsukuba 305-8602, Japan

**Keywords:** carbohydrate binding modules, cycloisomaltooligosaccharide, crystallography, glycoside hydrolase

## Abstract

*Paenibacillus* sp. 598K cycloisomaltooligosaccharide glucanotransferase (CITase), a member of glycoside hydrolase family 66 (GH66), catalyses the intramolecular transglucosylation of dextran to produce CIs with seven or more degrees of polymerization. To clarify the cyclization reaction and product specificity of the enzyme, we determined the crystal structure of PsCITase. The core structure of PsCITase consists of four structural domains: a catalytic (β/α)_8_-domain and three β-domains. A family 35 carbohydrate-binding module (first CBM35 region of *Paenibacillus* sp. 598K CITase, (PsCBM35-1)) is inserted into and protrudes from the catalytic domain. The ligand complex structure of PsCITase prepared by soaking the crystal with cycloisomaltoheptaose yielded bound sugars at three sites: in the catalytic cleft, at the joint of the PsCBM35-1 domain and at the loop region of PsCBM35-1. In the catalytic site, soaked cycloisomaltoheptaose was observed as a linear isomaltoheptaose, presumably a hydrolysed product from cycloisomaltoheptaose by the enzyme and occupied subsites –7 to –1. Beyond subsite –7, three glucose moieties of another isomaltooiligosaccharide were observed, and these positions are considered to be distal subsites –13 to –11. The third binding site is the canonical sugar-binding site at the loop region of PsCBM35-1, where the soaked cycloisomaltoheptaose is bound. The structure indicated that the concave surface between the catalytic domain and PsCBM35-1 plays a guiding route for the long-chained substrate at the cyclization reaction.

## Introduction

Cycloisomaltooligosaccharides (CIs) are a cyclic sugar chain consisting of α-1,6-linked glucose molecules [1] and several bacteria have known to produce CIs. CIs with degrees of polymerization of 7–17 (CI-7–CI-17, CI-*n*: CI with* n* degrees of polymerization of glucose) were found in *Paenibacillus* sp. 598K cultures when this bacterium was grown with dextran [2,3]. CIs are strong inhibitors of streptococcal glucansucrases and are expected to be useful for cariostatic food additives [4]. Among CIs, CI-10 was found to show inclusion ability prominently when it was tested by the colour stabilization of Victoria Blue B, suggesting that CI-10 may be useful for stabilizing unstable substances [5].

CI-producing bacteria produce CI glucanotransferase (CITase; EC 2.4.1.248), which catalyses the production of CIs from dextran by intramolecular transglucosylation, and also catalyses disproportionation, coupling and hydrolytic reactions [6]. CITases and some dextranases (EC 3.2.1.11; displaying endo-dextranolytic activity) are classified into the glycoside hydrolase (GH) family 66 (GH66) based on their primary structures [7,8], according to the CAZy database. These enzymes share 20–30% of amino acid sequence identity. GH66 dextranases produce only isomaltooligosaccharides (IGs), whereas CITases produce mainly CIs and small amounts of IGs. Currently, three 3D structures of GH66 enzymes have been determined and the enzymes have a modular protein architecture with a (β/α)_8_ TIM-barrel as a GH66 catalytic domain and several other β-domains [9–11]. Three domains, the catalytic domain and two β-domains on the N- and C-terminal ends, are conserved in most GH66 enzymes, and all the known CITases have extra β-domains. The crystal structure of CITase from *Paenibacillus agaridevorans* T-3040 (CITase from *P. agaridevorans* T-3040 (PaCITase), which was previously known as BcCITase from *Bacillus circulans* T-3040) revealed that a β-jellyroll domain belonging to the carbohydrate-binding module (CBM) family 35 (CBM35) is inserted in the catalytic domain at the seventh loop of the (β/α)_8_ TIM-barrel, and this domain protrudes from the catalytic domain and possesses two sugar-binding sites [10]. The first sugar-binding site is located on the variable loop region and recognizes the mid-point glucose molecule of α-1,6-linked glucans. The second site is located at the edge of one β-sheet, recognizing the non-reducing end glucose of α-1,6-linked glucans. The amounts of the different CIs produced by PaCITase follow the order (most to least): CI-8, CI-7, CI-9 and the longer CIs. Crystal structure of PaCITase has revealed that the bound isomaltooctaose (IG-8, IG-*n*: IG with* n* degrees of polymerization of glucose) in the catalytic site extends to the second sugar-binding site of CBM35, which acts as subsite –8, representing the enzyme–substrate complex when the enzyme produces CI-8 (Protein Data Bank (PDB) ID: 3WNN). This structure indicates that CBM35 functions to determine the size of the product, causing the predominant production of CI-8 by the enzyme.

Among CI-producing bacteria, the *Paenibacillus* sp. 598K strain showed good CI production and high CITase activity that was as good as the *P. agaridevorans* T-3040 strain [2]. *Paenibacillus* sp. 598K uses CITase (CITase from *Paenibacillus* sp. 598K (PsCITase)) to synthesize CIs from dextran. When the bacterium produces CIs from starch, it uses another enzyme α-1,6-glucosyltransferase (EC 2.4.1.-) to produce α-1,6-glucosyl-α-glucosaccharides from starch, which are used as substrates for PsCITase [12]. PsCITase is composed of 972 amino acids, which were deduced from the DNA sequence of the *cit* gene [3]. A domain search indicates that PsCITase has a modular structure consisting of at least five structural domains including two CBM35 domains, first CBM35 region of *Paenibacillus* sp. 598K CITase (PsCBM35-1) and PsCBM35-2, and the domain arrangement is similar to that of PaCITase. Although these two enzymes show a high sequence identity of 65%, the CI produced in the highest amounts by PaCITase is CI-8, whereas PsCITase produces CI-7 in the highest amount. Since the high CI-8 productivity of PaCITase is attributed to the sugar-binding site in the CBM35-1 domain working as subsite –8, we hypothesize that the structure of PsCBM35-1 might provide clues that clarify the mechanism of product specificity for PsCITase.

In the present study, the crystal structure of PsCITase we determined in its apo- and CI-7 complex forms, and structural differences between the CBM35 domains of PaCITase and PsCITase explain the substrate recognition mechanism of these enzymes that yields different product specificities. The elucidated sugar-binding mechanism of CITases would help in creating the functional enzymes to produce CIs with different polymerization degrees for certain purposes in industrial and material applications.

## Materials and methods

### Protein expression and crystallization of PsCITase

The expression plasmids coding for the core domain of the *cit* gene (41-739; N-terminal 40 amino acids and the C-terminal 235 amino acids were truncated) and constructed from the pET15b system (Merck-Novagen, Darmstadt, Germany) was transformed into *Escherichia coli* BL21(DE3) cells [3]. The cells were grown in LB medium (Merck, Darmstadt, Germany) containing 100 μM ampicillin at 37°C. Protein production was induced by the addition of isopropyl β-thiogalactoside to a final concentration of 1 mM followed by incubation of the cells at 25°C overnight. After *E. coli* cells were disrupted by sonication, PsCITase was purified to homogeneity by nickel-chelating chromatography using a His-Trap Fast Flow 5-ml column (GE Healthcare, Little Chalfont, U.K.). The purified sample was buffer exchanged and concentrated to 3.0 mg ml^−1^ by ultrafiltration using an Amicon Ultra-30000 MW membrane (Merck-Millipore, Billerica, MA, U.S.A.), and the concentration was checked during this process using the molar extinction coefficient (ε_280_ =1.62 × 10^5^ M^−1^ cm^−1^).

The protein solution was filtered through a 0.1-µm membrane (Merck-Millipore). PsCITase was crystallized by the sitting-drop vapour diffusion method at 293 K using a precipitant solution consisting of 25% PEG3350 (Hampton Research, Aliso Viejo, CA, U.S.A.) and 12% Tacsimate (Hampton Research), pH 5.0. Rod-type crystals with maximum dimensions of 0.05 × 0.05 × 0.3 mm appeared within a month using 50 μl of the reservoir solution with a drop consisting of 1 μl of protein solution and 1 μl of reservoir solution.

### Structure analysis of PsCITase

Diffraction experiments for protein crystals were conducted at the beamline BL-NW12 of the Photon Factory Advanced Ring (PF-AR), High Energy Accelerator Research Organization, Tsukuba, Japan [13]. The crystal was scooped in a nylon cryoloop (0.3 mm, Hampton Research) and then flash-cooled under a nitrogen stream at 95 K. Diffraction data by synchrotron radiation were collected at a wavelength of 1.0000 Å with a Quantum 270 CCD detector (Area Detector Systems Corp., Poway, CA, U.S.A.). The data were integrated and scaled using the programs DENZO and SCALEPACK in the HKL2000 program suite [14]. For the CI-7 complex data, 0.5 μl precipitant solution containing 5% CI-7 was added into the crystal drop for 3 min before data collection.

The crystal structure was determined by the molecular replacement method using the structure of PaCITase (PDB code: 3WNK, [9]) as the template structure using the program MolRep [15] incorporated in the CCP4 program suite [16]. Manual model building and molecular refinement were performed using COOT [17] and REFMAC5 [18]. Data collection and refinement statistics are shown in Table 1. Model stereochemistry was determined with the program RAMGAGE [19]. Structural illustrations were drawn with the program CueMol2 (http://cuemol.sourceforge.jp/en/).

**Table 1:** Data collection and refinement statistics of the PsCITase crystal structures

	Native PsCITase	PsCITase–CI-7 complex
PDB code	5X7G	5X7H
*Data collection*		
Space group	*P*3_1_21	*P*3_1_21
Cell parameters (Å)	*a* =113.7, *b* =113.7, *c* =121.6	*a* =112.8, *b* =112.8, *c* =122.3
X-ray source	PF-AR BL-NW12	PF-AR BL-NW12
Wavelength (Å)	1.00000	1.00000
Resolution (Å)	100–2.2 (2.28–2.20)*	100–2.6 (2.69–2.60)*
Number of reflections	1026298	581057
Unique reflections	46942 (4611)	28297 (2787)
Completeness (%)	100.0 (100.0)	99.9 (100.0)
Multiplicity	22.1 (22.1)	20.5 (19.4)
*R*-merge	0.096 (0.848)	0.179 (0.922)
Average I/σ	32.1 (4.9)	10.9 (3.3)
*Refinement*		
Resolution (Å)	98.5–2.2 (2.26–2.20)	97.7–2.6 (2.66-2.60)
*R*-factor	0.174 (0.254)	0.155 (0.255)
*R*-free	0.222 (0.334)	0.215 (0.306)
Number of water molecules	289	266
Average *B*-value (Å^2^)	45.4	44.4
r.m.s.d. from ideals		
Lengths (Å)	0.011	0.013
Angles (°)	1.43	1.64
Ramachandran plot (%) (favoured/allowed/disallowed)	96.1/3.7/0.1	95.4/4.4/0.1

^*^Values in parentheses refer to the highest resolution shell; r.m.s.d., root mean square deviation.

### Site-directed mutagenesis and enzyme assay of PsCITase

Site-directed mutagenesis was performed by polymerase chain reaction using KOD-plus DNA polymerases (TOYOBO, Osaka, Japan). The template was CITase-598K expression plasmid, which contains the N-terminal His_6_-tagged full-length *cit* gene without signal sequence cloned into pET-15b as described before [3]. The primer sets for each mutant are as follows: Y470A, 5′-GAGGGCGCCTATTCGCTTGTCTTCCGCTT-3′ (sense) and 5′-CGAATAGGCGCCCTCCTCCGGTACGGTG-3′ (antisense); W507A, 5′-CCGAGCGCGAGCGCCTGGTCGCACGAGA-3′ (sense) and 5′-CGCTCGCGCTCGGCTGATTTTGGAAC-3′ (antisense); W515A, 5′-AGACGGCGCATCAGGTGTATCTGACGCCAGGCA-3′ (sense) and 5′-CCTGATGCGCCGTCTCGTGCGACCAGG-3′ (antisense); Y519A, 5′-CAGGTGGCTCTGACGCCAGGCACGCAC-3′ (sense) and 5′-CGTCAGAGCCACCTGATGCCACGTCTC-3′ (antisense).

Wild-type and mutant PsCITase were expressed, purified and buffer exchanged as described above. CITase assays were performed by measuring the amounts of CIs produced from dextran as described previously [3]. Reactions were performed with appropriate amounts of enzyme and 2% dextran 40 (Amersham Biosciences) in 50 mM sodium acetate buffer (pH 5.5) at 40°C, and produced CIs that were measured by HPLC system (LC Workstation Class-VP, Shimadzu, Co., Kyoto, Japan) equipped with an evaporative light-scattering detector (ELSD-LT, Shimadzu, Co.) using a TSKgel Amide-80 column (4.6 × 250 mm; Tosoh, Tokyo, Japan) as described recently [20]. The protein concentrations of the purified enzymes were determined by measuring the absorbance at 280 nm with BSA (Sigma–Aldrich, St. Louis, MO, U.S.A.) as the standard.

## Results

### Overall structure of PsCITase

The crystal structures of core PsCITase and its complex with CI-7 (PsCITase–CI-7 complex) were determined by the molecular replacement method at 2.20 and 2.60 Å resolutions respectively. The crystallographic parameters are shown in Table 1. The structural model includes one protein molecule in the crystallographic asymmetric unit. The N-terminal 20 residues, which are derived from the expression and purification tag originating from the pET-15b vector (Met^21^–Met^40^), were not identified because of the lack of electron density. Apart from the sugar and water molecules, one calcium ion and two sodium ions were observed. Some malonate ions were also observed, which were included in the crystallization conditions.

**Figure 1:**
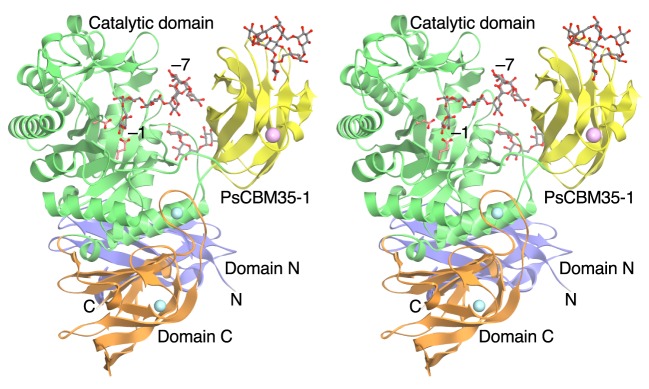
Stereo view of the ribbon model of the crystal structure of the PsCITase–CI-7 complex The bound CI-7 molecules are drawn as grey ball-and-stick models. The bound CI-7 at the catalytic site, which is hydrolysed by the enzyme to be linear IG-7, is bound at subsites –1 to –7 from the reducing end to the non-reducing end. Four domains are coloured as follows: the catalytic domain in green; the N-domain in blue; the C-domain in orange and the PsCBM35-1 domain in yellow. Two catalytic residues are indicated as pale red stick models near the subsite –1. Calcium and sodium ions are shown as pink and cyan spheres respectively.

The PsCITase–CI-7 complex structure is shown in Figure 1. The core PsCITase structure is composed of four domains, the catalytic domain and three β-domains. Domain N (Ala^41^–Ser^140^) composed of seven β-strands forms an Ig fold. The catalytic domain (Domain A, Asp^141^–Ile^420^ and Thr^548^–Asp^614^) comprising the (β/α)_8_-barrel is well conserved in GH66 enzymes. In the catalytic domain, the PsCBM35-1 domain is inserted as domain B (Gly^421^–Gly^547^) at the seventh loop of the (β/α)_8_-barrel. Domain C, positioned at the C-terminal side of the catalytic domain (Pro^615^–Met^738^), comprises a β-sandwich structure and is composed of Greek key motifs. After the C-terminal of domain C, one CBM35 domain (CBM35-2) and an extra function – unknown peptide exist in the deduced sequence of the *cit* gene; however, they have been removed for the stable protein expression [3].

**Figure 2:**
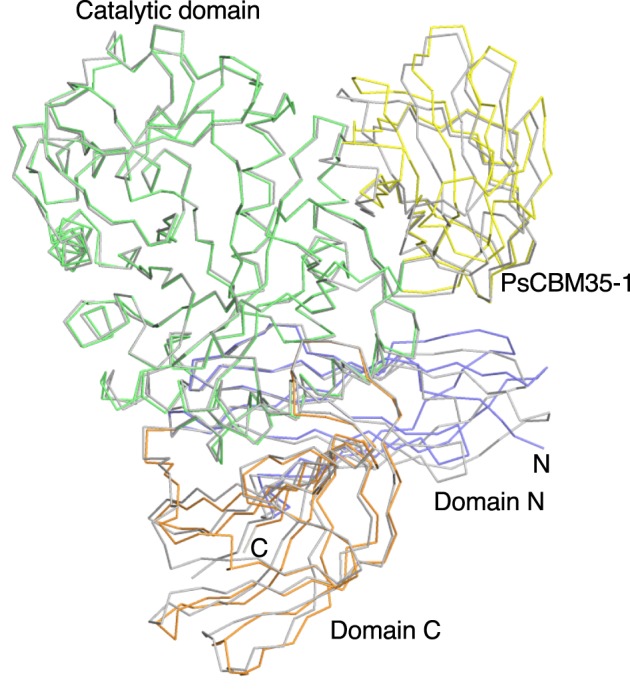
Superimposition of the Cα traces of the three core domains of GH66 CITases Wild-type PsCITase is shown in the same domain colours as in Figure 1 and PaCITase (PDB code: 3WNK, [10]) is shown in grey. Cα-traces are shown from the N-terminal domain N to the C-terminal domain C.

**Figure 3:**
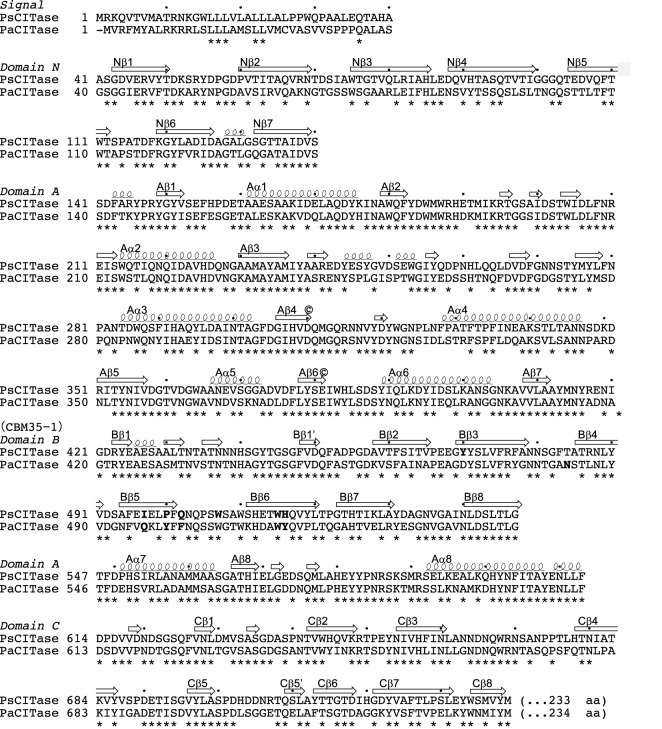
Structure-based amino acid alignment of the core domains of GH66 CITases The secondary structure elements of PsCITase are indicated by coils for α-helices, small coils for 3_10_-helices and arrows for β-strands. Residues conserved between the two sequences are marked below by asterisks. Catalytic residues are located at the ends of the fourth and sixth β-strands and indicated by ©. PsCBM35-1 residues in contact with the bound CI-7 molecule or PaCBM35-1 residues in contact with the bound IG-8 molecule are shown as bold fonts. The amino acid numbers of PsCITase are indicated by dots every 10 residues.

The overall structure is conserved with that of GH66 enzymes (Figures 2 and 3). The Cα-trace of PsCITase superimposed with that of PaCITase (PDB code: 3VMN, 65% amino acid identity [10]) gives an r.m.s.d. of 1.20 Å (Figure 2). When only the catalytic domains were superimposed, the r.m.s.d. resulted to be 0.49 Å, but the other three β-domains were rotated with their hinge region as an origin. In relation to the catalytic domain, the PsCBM35-1 domain was rotated ∼10° in a clockwise direction when compared with that of the CBM35 domain of PaCITase (first CBM35 region of *Paenibacillus agaridevorans* T-3040 CITase (PaCBM35-1), formerly BcCBM35-1) [10]. Domain rotations were also observed for the N- and C-domains with rotation values of ∼5°. The multivalent domain architecture of CITases appears to yield flexibility between domains, and the concave region between the catalytic domain and CBM35-1 can be used as the substrate-binding groove, as discussed later.

### CI-7 complex structure of PsCITase

**Figure 4:**
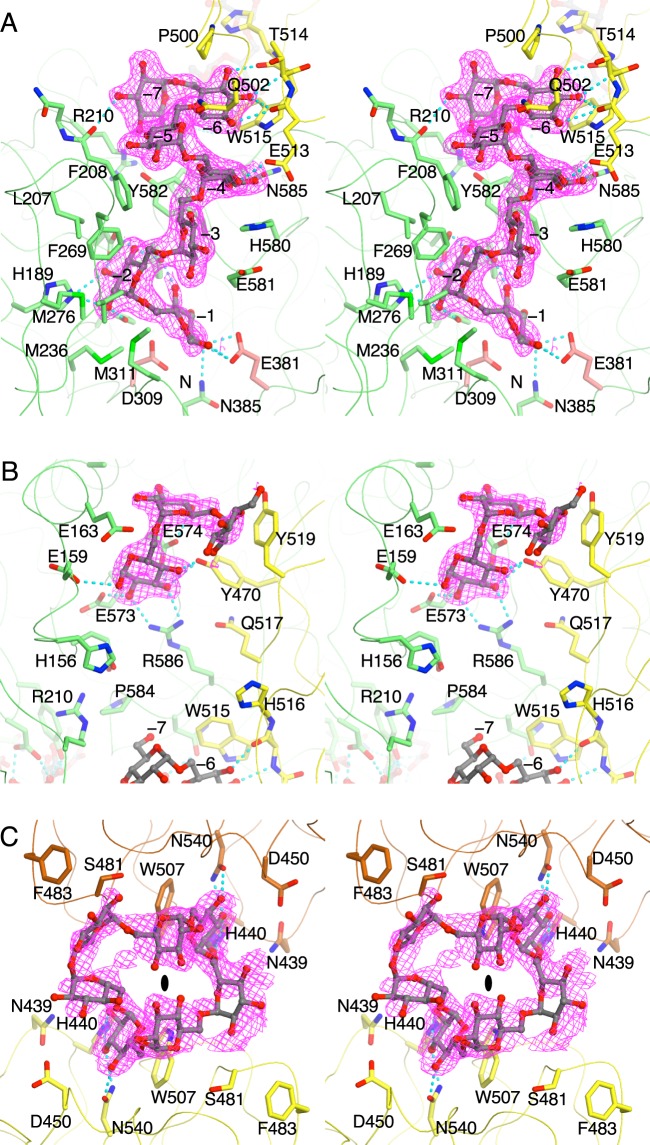
Stereoview of the structure of the soaked CI-7 molecules in PsCITase (**A**) The bound IG-7 molecule, which is the hydrolysis product of CI-7, in the catalytic cleft overlaid on to the PsCBM35-1. The 2Fo–Fc electron density map around the bound molecule is contoured at 1.0 σ and shown in magenta. The bound IG-7 molecule is shown as the ball-and-stick model and the surrounding residues are shown as stick models. The hydrogen bonds between IG-7 and protein atoms are shown as cyan dashed lines. (**B**) The bound short IG, which is derived as the hydrolysis product, between the catalytic domain and PsCBM35-1 near its joint area. (**C**) The bound CI-7 molecule in the variable loop site of PsCBM35-1. The CI-7 molecule is sandwiched between the crystallographic symmetry related two molecules (coloured in yellow and brown) and located on the crystallographic two-fold symmetry shown by the ellipse. The shown electron density presented is averaged by the symmetry and multiple conformations.

In the complex structure with CI-7, CI-7 and IGs were observed at three sites (Figures 1 and 4), in the catalytic cleft, at the joint of the PsCBM35-1, and at the loop region of PsCBM35-1.

At the catalytic site, the bound CI-7 was observed as the cleaved linear form like isomaltoheptaose (IG-7) in the catalytic cleft (Figure 4A). Electron density corresponding to seven glucose moieties was observed. The reducing end of the resultant IG-7 was located at subsite –1 and in close proximity to the catalytic residues of PsCITase, Asp^309^ and Glu^381^, and the sugar chain elongated into the gap between the catalytic domain and the CBM35-1 domain with a zigzag chain structure owing to the α-1,6 glucosidic linkages. According to the subsite nomenclature for GHs [21], the position of the reducing end glucose is designated as subsite –1, the position of the seven glucose moieties from the reducing end (Glc–1 to Glc–7) are designated as subsites –1 to –7. The four glucose moieties Glc–1 to Glc–4 were located along the catalytic cleft, and occupied four subsites –1 to –4, which is a common structural feature of other GH66-enzymes solved previously [9–11]. Subsites –4 to –7 were located between the catalytic and CBM35-1 domains. Glc–4 was in contact with both domains by polar interactions, whereas Glc–5 was loosely sandwiched by hydrophobic contacts. Glc–6 was stacked by the side chain of Trp^515^ and hydrogen-bonded by the main chain of the sixth β-strand of CBM35-1 domain. Glc–7 formed a hydrogen bond with the main chain of Phe^208^ of the catalytic domain. Glc–2 to Glc–7 had the normal ^4^*C*_1_ chair conformation, but the electron density for the Glc–1 was rather ambiguous and Glc–1 was refined to have an unusual *E*_3_ conformation [22].

A short IG was found between the catalytic and CBM35-1 domains, besides the aforementioned IG-7 (Figure 4B). Three glucose moieties were observed, but two of them from the non-reducing end had ambiguous electron density. The main interaction involved hydrogen bonds between the reducing-end glucose and Glu^159^, Glu^573^ Asp^574^ and Arg^586^ side chains of the catalytic domain and the side chain of Tyr^470^ of the CBM35-1 domain. The non-reducing end glucose was stacked on the side chain of Tyr^519^. This sugar chain may form part of the hydrolysed product of CI-7 by the active enzymes.

The third bound ligand was observed in the canonical sugar-binding site of the CBM35-1 domain (Figure 4C), as observed previously in the PaCITase–ligand complex [10]. The manner in which the sugar binds is mostly conserved between PsCITase and PaCITase. The bound sugar appeared to be soaked CI-7 with the cyclic sugar chain structure, but the electron density was partly ambiguous because the bound sugar ligand was located on the two-fold axis of the crystal. One CI-7 molecule was bound between the two-fold symmetry related two molecules and Trp^507^ from both molecules stacked two glucose moieties of the bound CI-7. However, the CI-7 molecule did not have two-fold symmetry in its structure; the bound structure took multimodal conformations. The electron density was averaged by the symmetry between the multimodal conformations and this resulted in indistinct density. The Trp^507^ side chain functioned as a platform for stacking interactions and the surrounding residues formed hydrogen-bonding interactions. These interactions provided the binding site specific to the mid-point glucose of the α-1,6-glucosyl linkage.

**Figure 5:**
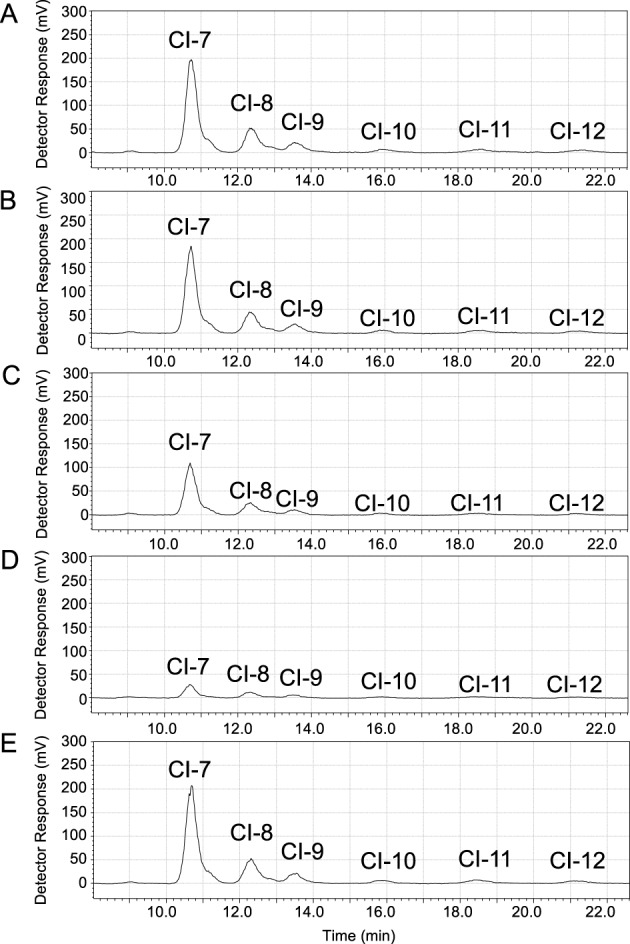
HPLC analysis of CIs produced by wild type and mutant PsCITases (**A**) Wild-type PsCITase, (**B**) Y470A mutant, (**C**) W507A mutant, (**D**) W515A mutant and (**E**) Y519A mutant. Each enzyme solution (32 nM) was incubated in 50 mM sodium acetate buffer, pH 5.5 containing 2% dextran 40 for 15 min at 40°C. The CIs produced were analysed by HPLC. Details of the procedures are described in the ‘Materials and methods’ section.

To investigate the role of the sugar-binding site of PsCITase, four mutants that changed the aromatic side chains at the sugar-binding sites of PsCBM35-1 were prepared. The mutants were W515A (subsite –6), Y470A, Y519A (these two residues were in the concave between the catalytic domain and CBM35-1) and W507A (canonical sugar-binding site of CBM35-1). As shown in Figure 5, the CI-production pattern by these mutants was measured using dextran 40 as a substrate. Overall CI production of the W507A and W515A mutants was reduced approximately by 25 and 65% comparing with the wild-type enzyme respectively, whilethat of Y470A and Y519A retained the activity. All the mutants mainly produced CI-7 and showed the same characteristics with wild-type enzyme.

## Discussion

The obtained structure of the protein–sugar complex was prepared by soaking CI-7 into the crystal drop. The expected structure of the complex should have contained the docked cyclic IGs in the catalytic site. However, the hydrolysed linear IGs were present in the structure, enabling us to identify the subsites from the catalytic site to the CBM35-1. The identified subsites indicated that the concave region formed by the catalytic domain and CBM35-1 supply the substrate-binding site for dextran, the long chain substrate.

**Figure 6:**
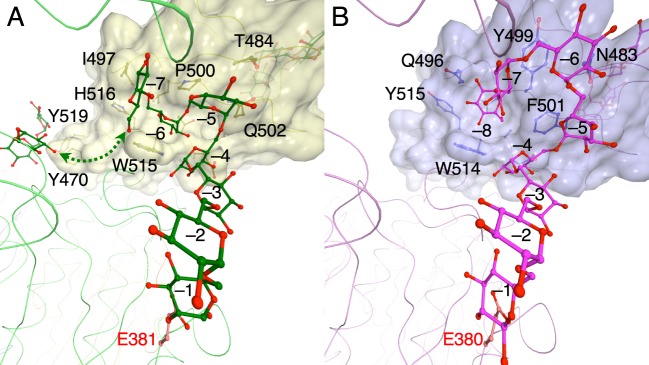
Structural comparison between the PsCITase–CI-7 complex and the PaCITase–IG-8 complex (**A**) PsCITase–CI-7 complex. **(B)** PaCITase–IG-8 complex [10]. Figures are viewed from the catalytic site. The bound sugars are shown by ball-and-stick models. CBM35 domains are shown in surface model and the Cα-trace of the catalytic domain is shown by wire drawings.

The PsCITase–CI-7 complex structure and the PaCITase–IG-8 complex structure [10] both showed that the sugar chains dock from the catalytic site to the CBM35-1 domain; however, the manner in which the sugar binds at the conserved tryptophan, Trp^515^ in PsCITase and Trp^514^ in PaCITase, in the CBM35-1 domain differed (Figure 6). In PsCITase, the Trp^515^ side chain provides the platform for parallel stacking on the glucose moiety as subsite −6 and recognizes the mid-point glucose moiety of the α-1,6-glucan. The C1-and C6-hydroxyl groups of the bound glucose moiety were positioned at both sides of the platform to form α-1,6-glucosyl bonds with the adjacent glucose moieties (Figure 6A). On the other hand, the PaCITase Trp^514^ side chain participates by forming the side wall of the binding pocket, which functions as subsite –8, along with the PaCITase Tyr^499^ and Phe^501^ residues, and the C6-hydroxyl group of the bound glucose moiety is buried at the bottom of the pocket, concomitantly expressing the terminal-binding mode (Figure 6B). The surface drawing of the binding sites demonstrates that the subsite –6 of PsCITase is shaped like a narrow cleft, whereas the subsite –8 of PaCITase is shaped like a shallow pocket. The non-conserved amino acids, Pro^500^, Gln^502^, Ile^497^ and His^516^, of PsCITase contribute to the glucose-binding mode.

Comparison of the IG structures docked in these two enzymes also showed that the subsite location from –1 to –4 are conserved in these enzymes, but the subsite positions from subsite –5 to −8 differ (Figure 6). The different positions at subsite –5 might be caused by substitution of the amino acid at Gln^502^. In PaCITase, the bulky Phe^501^ side chain in PaCITase forms the sugar-binding pocket and creates steric hindrance. Thus, the IG-8 sugar chain binds in a manner that circumvents Phe^501^. On the other hand, the Gln^502^ side chain of PsCITase adopts a conformation that enables a shortcut for the substrate to bind between the catalytic site and the binding site at Trp^515^. This different conformation of Gln^502^ means that the conserved Trp^515^ of PsCITase forms the proximal subsite −6, whereas PaCITase Trp^514^ is involved in subsite −8. Thus, the different product specificity of these two CITases could be attributed to the structural difference at the second sugar-binding site of CBM35-1. Since PsCITase does not have the terminal-binding subsite –8, PsCITase does not produce CI-8 predominantly, but produces CIs with degrees of polymerization of seven or higher, with the polymerization degree seven being the main product by PsCITase and higher polymerization degrees being produced at lower amounts as the polymerization length increases. The alanine mutation on Trp^515^ did not affect the product specificity of the enzyme but reduced the overall production of CIs, and Trp^515^ appeared to function as a component of substrate-binding cleft for the long substrate.

In the direction beyond the subsite −7, three glucose moieties, which appeared to be a part of IGs produced by hydrolysis of soaked CI-7, were observed. The α-anomeric hydroxyl group of the reducing-end glucose that is located at the inner position of the concave is directed towards Glc–7 of the docked IG-7. No electron density was observed between the two IG sugar chains, but the concave seemed wide enough for IGs to cover both docked sugar chains. The distance between the Glc–7-O6 atom and the reducing end O1 atom of the docked sugar chains was ∼12 Å, and at least three glucose molecules might be necessary to link the two docked sugar chains, indicating that the positions of the newly found three glucose moieties corresponded subsites –11 to –13. Although these sites did not affect CI productivity and size specificity of the enzyme, the concave region between the catalytic and PsCBM35-1 domains appears to be a good guide route for a substrate with a long sugar chain, such as dextran, to access the catalytic site.

In our previous study on PaCITase, we succeeded in producing engineered PaCITase mutants that produce large amount of CIs with higher degrees of polymerization by introducing mutations around the IG-binding subsites [20]. The success in this mutational study indicates that the mutations might enable us to change the degrees of polymerization of the CIs produced for particular purposes. CIs form inclusion complexes and this depends on the degrees of polymerization. Production of CIs of certain degrees of polymerization should facilitate formation of chemical-inclusion complexes and based on structural data of these enzymes, the required CIs may be produced by particular CITase mutants.

CBMs are usually small domains and more than 30 CBM families adopt a β-sandwich fold. In the CBMs comprising the β-sandwich structure, sugar-binding sites are located mainly at two sites: the major site is termed the variable loop site and is located on the tip of the β-turn, whereas the other binding site is located on the surface of the concave β-sheet and is termed the concaved face site [23]. CBM35s adopt a conserved β-sandwich structure with a β-jellyroll motif and one conserved calcium-binding site. However, CBM35s show variable ligand specificity and are classified into four major subfamilies I–IV based on their ligand specificities [24], and the representative sugar binding occurs at the variable loop site. PsCBM35-1 and PaCBM35-1 have conserved first sites that bind to the mid-point glucose of the α-1,6-glucan, and are grouped into the subfamily III, that recognizes glucosyl oligosaccharides. The role of the first site of PsCBM35-1 is substrate binding to increase catalytic efficiency. The W507A mutant showed a certain decrease in CI production. On the other hand, the second binding site of PsCBM35-1 was located at the side of two β-sheets and is not part of the concave face site. In this case, the second site in PsCBM35-1 could be considered a lateral edge site. At this second site, PsCITase recognizes the mid-point glucose of substrate, long substrate chains like dextran and supports the cyclizing reaction of the enzyme by guiding the substrate to the catalytic site.

We have determined the crystal structure of PsCITase, elucidating IG-binding mode at the catalytic site and PsCBM35-1. Two sugar-binding sites in PsCBM35-1 did not affect size specificity of the produced CIs, but enhanced the total CI production of the enzyme. The concave between the catalytic domain and PsCBM35-1 appeared to be a guiding route for the long-chained substrate. The information obtained here provides the structural basis for enzymatic improvement of PsCITase.

## Footnotes

The atomic co-ordinates for the sugar-free PsCITase and its complexes with CI-7 have been deposited in the PDB (http://www.pdb.org/) under code 5X7G and 5X7H.

## Abbreviations

GH: glycoside hydrolase
GH66: glycoside hydrolase family 66
CBM: carbohydrate-binding module
CBM35: carbohydrate-binding module family 35
CI: cycloisomaltooligosaccharide
CI-*n*: cycloisomaltooligosaccharide with *n* degrees of polymerization of glucose
CITase: cycloisomaltooligosaccharide glucanotransferase
IG: isomaltooligosaccharide
IG-*n*: isomaltooligosaccharide with *n* degrees of polymerization of glucose
PaCBM35-1: first CBM35 region of *P. agaridevorans* T-3040 CITase
PaCITase: CITase from *P. agaridevorans* T-3040
PDB: Protein Data Bank
PsCBM35-1: first CBM35 region of *Paenibacillus* sp. 598K CITase
PsCITase: CITase from *Paenibacillus* sp. 598K
r.m.s.d.: root mean square deviation (difference)
